# Assessment the knowledge, care, and experiences of neonatal nurses about enteral nutrition

**DOI:** 10.1097/MD.0000000000031081

**Published:** 2023-05-26

**Authors:** N. Ecem Oksal Gunes, Senay Cetinkaya

**Affiliations:** a Cukurova University, Health Sciences Institute, Adana, Turkey; b Cukurova University, Faculty of Health Sciences, Department of Nursing, Child Health and Diseases Nursing (Associate Professor), Adana, Turkey.

**Keywords:** enteral nutrition, nurse, nursing, neonatal

## Abstract

Enteral-feeding refers to any nutritional method throughout the gastrointestinal tract, including oral feeding. This qualitative study examined the information, experiences, and records of neonatal nurses of enterally fed patients. The study was conducted between 05.04.2018 and 05.05.2018 with 22 nurses (73.3%) working in the neonatal intensive care clinic of Çukurova University Balcali Hospital, Adana, Turkey. The data were collected by “Observation and Interview Form” developed based on the literature. Nurses were observed, and interviews were conducted depending on their appointments. Data were collected by observing each nurse on 2 different days. In all observations; it was determined that the nurses changed the feeding set daily, regularly checked the location of the feeding tube and amount of residue, and administered medication through the feeding tube. In 22.7% of the observations, nurses did not securely fix the feeding tube, 27.2% did not write a daily date on the injector with the residual volume measured, and 31.8% did not wash the injector. All the nurses recorded the amount of feed, residual amounts, and content. At the end of the interviews, 9% of the nurses stated that they had experienced aspiration among the complications encountered during enteral feeding. During the interview, they stated that all nurses were educated about enteral nutrition, had control of whether the probe was in place before feeding, performed residual control, washed their hands before the procedure, fixed the food injector to 1 place, and allowed the food injector to flow spontaneously with negative pressure. According to the results of the interviews and observations, nurses could not reflect on their nursing practices correctly. Nurses working in neonatal intensive care units should be regularly trained to share the results of evidence-based studies on enteral nutrition.

## 1. Introduction

Globally, an estimated 15 million babies are born before the 37th week of pregnancy each year, indicating that they are born prematurely or preterm. In 184 countries, the preterm birth rate ranges from 5% to 18% of babies born.^[1]^ Most patients require special care in the neonatal intensive care unit until they can maintain their nutritional and respiratory needs. Nutrition is the basic requirement for newborns to achieve high growth, development, survival, and health protection.^[2]^ Appropriate and effective enteral nutrition reduces morbidity and mortality rates in newborns. The most important problems encountered in enteral nutrition in newborns at risk are late recognition of nutritional needs, failure to follow enteral nutrition protocols, and errors in medical practice (such as incorrect connections).^[3]^

A frequently encountered problem is the need for information from health professionals regarding enteral feeding. The role of nurses in enteral nutrition varies according to hospital policies. These roles include attaching the nasogastric (Ng) feeding tube to the patient, maintaining the feeding tube, providing the recommended nutrients to the patient, preventing complications, identifying and interpreting changes in the patient in the early period in case of complications, and informing the physician by taking the necessary precautions.^[4,5]^ Preventable complications can develop if nurses are unable to provide appropriate care to enterally fed patients. Therefore, conducting enteral nutrition practices according to evidence-based practices is extremely important for improving the quality of nursing care. There are a sufficient number of studies on enteral nutrition in the literature. Studies have shown that nurses do not have sufficient knowledge of enteral nutrition practices, precautions against possible complications are not taken at a sufficient level, and they should be supported with in-service training. Bedier et al (2016) reported that education on enteral nutrition provided to nurses increased their knowledge and working skills.^[6]^ A similar study, reported that the knowledge level of nurses was insufficient before training on enteral nutrition in the neonatal intensive care unit.^[7]^ Nurses should provide care in line with evidence-based recommendations through training to reduce the morbidity and mortality rates of enterally fed newborns. This study aimed to examine neonatal nurses’ knowledge and experience about enteral nutrition.

### 1.1. Enteral nutrition

Enteral nutrition involves the administration of nutrients via oral, orogastric (og)/Ng, and transpyloric catheters. The preferred method is determined by the gestational age of the newborn, clinical condition, and oral feeding ability. Therefore, alternative nutritional methods should be used.^[8,9]^ Enteral nutrition is used in infants with endotracheal intubation or in those who are immature, weak, and without sucking, swallowing, or gagging reflex.

Foods that can be provided to newborns through enteral nutrition include mother’s milk, enriched breast milk, pasteurized donor milk, and enteral diet formulas.^[10]^ The American Academy of Pediatrics, World Health Organization, and many nutrition committees have stated that the first choice of food in the feeding of preterm babies is breast milk.^[11,12]^ The first administration of colostrum for milking is important for the development of the baby. Nurses should constantly support mothers using breast milk during the prenatal period.^[2]^

Minimal enteral nutrition, or a hypocaloric diet/initial diet, involves very low nutrient volumes (10-20 cc/kg/day). It was initially administered to acclimate the gastrointestinal system to food, stimulate digestive hormones, and accelerate gastrointestinal system maturation. Feeding was started at the 48th hour and continued at the initial level for the first 1 to 2 weeks. It is found that the initial feeding is well tolerated. It has been shown to increase bone mineralization in very low birth weight infants by reducing indirect hyperbilirubinemia and cholestatic jaundice.^[10]^ Minimal enteral nutrition should be provided with breast milk, and breast milk enhancers should be added after starting to meet half of the nutritional requirements of breast milk. Another recommendation is to start breast milk enhancers when the daily enteral intake is 100 mL/kg/day.^[11]^

The gavage feeding method involves feeding food through the mouth or nose to the stomach, using a polyethylene catheter. It is applied to babies who are less than the thirty-fourth gestational week with insufficient sucking and swallowing reflexes, who get tired quickly during feeding, and who cannot be fed orally because of severe neonatal diseases or congenital malformations with high respiratory rates.^[13]^ Probe placement and measurement should be performed carefully. Before feeding, the stomach contents were checked and recorded. The risk of gastroesophageal reflux can be reduced by removing ogs/ngs between feeding sessions. Changing the catheter may cause apnea and bradycardia, as it increases vagal stimulation. The probe may be left in place, as some babies may develop extreme intolerance to the removal and insertion of ogs/ngs. The thickness of the feeding probes was chosen in proportion to the baby’s weight. Probe number 3.5 to 5 was used for babies weighing <1000 g, and probe number 5 to 8 were used for babies weighing ≥1000 g. Babies weighing less than 1000 g were fed at 2-hour intervals, while babies weighing over 1000 g were fed at 3-hour intervals.^[8]^ In general, there is insufficient evidence regarding the difference between 2-hour and 3-hour feedings for infants weighing ≤1250 g.^[14]^

Application of Nutrition Probe

• Materials are prepared.
• Nurses should wash their hands.
• Gloves are worn after providing hand hygiene.
• The baby is placed on his back.
• Non-nutritive suction is initiated.
• The orogastric probe was measured from the midline of the lip to the earlobe and lower end of the xiphoid.
• The Ng probe was measured and marked from the tip of the nose to the earlobe and the xiphoid.
• While the probe was applied to the baby, it was gently inserted through the nose to the marked place.
• A syringe was attached to the tip of the probe and the stomach contents were aspirated. The quality and amount of incoming content were recorded and returned.^[3]^ The excretion of stomach contents during each feeding period leads to alkalosis.
• In very small premature babies, 1 mL of air is injected through the catheter, and the sound of entering the stomach is listened to with a stethoscope.
• After determining that the probe was in the right place, fixation was performed.
• Feeding is started with gavage.
• The position of the probe is checked before each feeding.
• The breast milk/formula drawn into the syringe should be held 20 cm above the baby to allow gravity drainage of the formula into the stomach.^[15–19]^

The points that the nurse should pay attention to during feeding are as follows:

• Before starting feeding, whether the catheter is in the stomach should be checked,
• Residue control should be made,
• Food should not be given under pressure using the injector plunger,
• The flow rate of the food should not exceed 5 mL in 5/10 minutes in premature babies and infants.
• Feeding should be initiated in small amounts, and the amount should be increased as the baby tolerates.
• The food prepared should be at room temperature,
• If the probe remains in place where it will not be pulled, the inside of the probe should be cleaned by giving 1 to 2 mL sterile water after feeding.
• The amount of food given after feeding, and, the time and position of the baby were recorded.
• The frequency and amount of vomiting, regurgitation, abdominal distention, and stool should be recorded to determine nutritional tolerance.^[16]^

## 2. Methods

This study was conducted through observation and interviews using qualitative research methods that examined the knowledge, experience, and records of neonatal nurses of enteral-fed patients.

The research was planned with 30 nurses working in the neonatal intensive care units of Çukurova University Hospital Balcali Hospital, but 8 nurses did not allow observations and interviews. Data were obtained from 22 (73.3%) nurses.

### 2.1. Inclusion criteria of the study

Volunteer nurses who worked in the neonatal intensive care service between 05.04.2018 and 05.05.2018 with only day shifts (8.00-16.00) were included in this study.

### 2.2. Data collection techniques and data collection tools

For data collection, the “Observation Form,” developed by researchers based on the literature, was obtained using an interview method, which is a qualitative research methods.^[20–24]^

To test the content validity of the observation form prepared by the researchers, the questionnaire was submitted to an expert comprising 2 academics, 2 neonatal subspecialists, and 3 clinical nurses.

There were 18 items on the observation form, 12 items on applications related to enteral nutrition, and 6 items on registration.

The interviews were conducted through appointments and audio recordings. These voice recordings were listened to, and, the interviews were transcribed and deleted.

### 2.3. Application of data collection forms

All observations were made between 05.04.2018 and 05.05.2018 during the daytime, working hours (8.00-16.00), and by the same researcher. Data were collected by observing each nurse on 2 different days. The number of enteral-fed patients that nurses cared for during the day ranged from 3 to 6. Care practices in each enteral-fed patient were considered observations. A total of 216 observations were made at the end of the data-collection process.

During the interviews with the nurses, they were asked to explain what they did in enteral feeding practice step-by-step, the issues they should pay attention to, and what they should record on the nurse follow-up form.

Data for the records were collected from nurse follow-up forms after care applications were completed.

### 2.4. Data analysis

Data were analyzed from 216 observations of 22 nurses. Frequencies and percentages were determined using the SPSS demo package.

### 2.5. Ethical aspects of research

The study was conducted after obtaining informed consent from nurses. Ethics committee approval was obtained from the Faculty of Medicine Non-Interventional Clinical Research Ethics Committee of Çukurova University.

## 3. Results

### 3.1. The observational results of the nursing practices related to enteral nutrition are presented in Table 1.

As in the application of nutrition probe instruction, to which the nurse should pay attention before starting feeding, it should be checked whether the probe is in the stomach. In this study, 7.3% of the nurses fixed the feeding tube securely, and all nurses checked the tube location before feeding the newborn. It was observed that 86.4% of nurses changed their feeding set daily and wrote the date on it. It was determined that 72.8% of nurses wrote about the date of daily use of syringes for gastric residue control. It was observed that 68.2% of nurses left the injector clean after washing. All nurses checked the gastric residue before each feeding, washed the tube after administering medication from the feeding tube, gave the food to the patient at room temperature, and properly stored the remaining food in the bottle/box. It was observed that 93.1% of the nurses raised the patient’s head 30 to 45 during feding, and 88.8% of them gave food from the feeding tube at an appropriate rate.

**Table 1:** Observation results of nursing practices related to enteral nutrition (n = 216).

Nursing practices	Done		Not done	
	n	%	n	%
Securely fixing the feeding tube	167	**77.3**	49	22.7
Changing the nutrition kit daily and writing dates on it	186	**86.4**	30	13.6
Writing the daily usage date on the syringe used for gastric residue control	157	**72.8**	59	27.2
Checking the tube location before giving food and medicine	216	**100.0**	0	0.0
Checking gastric residue before each feeding	216	**100.0**	0	0.0
Washing the syringe and leaving it clean	147	**68.2**	69	31.8
Raising the patient’s head 30°-45° high during feeding	201	**93.1**	15	6.9
After giving nutrients and medicine, giving 1-2 mL sterile water through the feeding tube to clean.	216	**100**	0	0.0
Properly storing the food left in the bottle and the box	216	**100**	0	0.0
Feeding the food at the appropriate rate through the feeding probe	192	**88.8**	24	11.2
Giving the food to the patient at room temperature	216	**100**	0	0.0

The Observation Results of the Nurses on the Nurse Follow-up Records of Enteral Nutrition are shown in Table 2.

**Table 2:** Nurses’ registration on nurse follow-up form regarding enteral nutrition.

Data	Recorded		Not recorded	
	n	%	n	%
The amount of food and water given to patients	216	100.0	0	0.0
Gastric residual volume amount	216	100.0	0	0.0
Nutrition tube insertion date	188	87	28	13
Nutrition set replacement date	157	72.7	59	27.3
Registration of the remaining part of the Nutrition tube	68	31.8	148	68.2
Nutrition tube number	0	0.0	216	100.0

As in the application of nutrition probe instructions, the nurse should record the amount of food given after feeding, and, the time and position of the baby. In this study, all nurses recorded the amount of food given to the patients and the amount of residual gastric volume in the follow-up form. It was observed that 87% of the nurses recorded the date of feeding tube insertion, 72.7% of the nurses recorded the feeding set change date, and 31.8% of the nurses recorded the outside part of the feeding tube. In the observations, it was seen that none of the nurses recorded the feeding tube number.

### 3.2. The results of interviews related to nursing experiences on enteral nutrition are shown in Table 3.

All the nurses in the study stated that aspiration and vomiting may be among the complications that occur during application. Twenty nurses (90.0%) stated that necrotizing enterocolitis (NEC) may be a complication that may occur during application. Twelve nurses (54.5%) stated that perforation could be a complication that may occur during application. In addition, 63.6% of nurses stated that they experienced complications during their application. Regarding complications, 13.6% of the nurses stated that they experienced NEC, 81.8% experienced aspiration, and 90.9% experienced vomiting. During the interviews, it was observed that there were no nurses who experienced perforation during feeding.

**Table 3:** Interview report on nursing experiences related to enteral nutrition (n = 22).

Data		n	%
What are the complications that may occur during the application?	NEC	20	90.9
	Aspiration	22	100
	Vomiting	22	100
	Perforation	12	54.5
Does the nurse have any experience of complications that may occur during the application?	Yes	14	63.6
	No	8	36.4
Application-related complications	NEC	3	13.6
	Aspiration	18	81.8
	Vomiting	20	90.9
	Perforation	0	0.0

### 3.3. The results of interviews with nurses on enteral nutrition considerations are shown in Table 4.

Giving the food at room temperature, not giving food under pressure, checking the tube location by giving 1 mL of air and listening the sound of entering the stomach with a stethoscope before feeding, cleaning by giving 1 to 2 mL of sterile water after feeding are the issues that nurses should pay attention to. In the interview, all nurses stated that they took food from the refrigerator 30 minutes to 1 hour beforehand and gave it to the baby when it reached room temperature. Sixteen nurses (72.7%) stated that they checked the tube location by looking at the residue, and 27.3% checked the tube location by providing air from the tube and listening with a stethoscope. Twenty nurses (90.9%) stated that they changed their feeding set daily, and 15 nurses (68.1%) wrote about the date of daily use of the injector. All nurses stated that they checked the gastric residue before each feeding and left the residual injector clean by washing it after use. All nurses stated that they checked the location of the tube before each feeding, changed the patch if it did not seem intact, and sent 1 to 2 mL of sterile water to clean the tube after providing food or medicine. A total of 90.9% of the nurses stated that they had kept the food box in the refrigerator after opening. Twenty-one of the nurses, 95.4% stated that they raised their head by 30° to 45° during feeding. Eighteen of the nurses, 81.8% stated that they expected food to flow with negative pressure by removing the plunger of the injector during feeding. A total of 18.2% of nurses stated that they sent food slowly from the tube with an injector.

**Table 4:** Interview report with the nurses on the issues to be considered in enteral nutrition (n = 22).

Themes	Theme sets	n	%
Giving the food at room temperature	I take out the food in the refrigerator 30 min to 1 h in advance.	22	100
Checking the tube location before giving food and medicine	I can see if the tube is in place by looking at the residue.	16	72.7
	I can understand by giving air and with a stethoscope.	6	27.3
Frequency of change of nutrition set	I replace it if the probe is not in place and if it looks dirty and intact.	2	9.1
	I change it daily.	20	90.9
Application of gastric residue control	I check gastric residue before each feeding.	22	100
	I wash the residue injector and leave it clean	22	100
	I write the daily use date on the syringe.	15	68.1
Securely fixing the nutrition tube	Before each feeding, I check the location of the tube and replace it if it does not seem intact.	22	100
Raising the patient’s head 30°-45° high during feeding	Before feeding the baby, I raise her head with the pillow.	21	95.4
Giving water after giving nutrients through nutrition tube	After giving the formula or medicine, I give 1-2 mL sterile water to clean the tube.	22	100
Properly storing the food left in the bottle and box	After the food box is opened, I store it in the refrigerator.	20	90.9
	Until next feeding, I keep it in an incubator.	2	9.1
Giving the food at the appropriate rate through the feeding probe	By removing the plunger of the syringe, I wait for the food to flow with negative pressure.	18	81.8
	I send the food slowly through the tube with the injector.	4	18.2

The Results of the Interviews Regarding the Nurse Follow-up Records of Enteral Nutrition are shown in Table 5.

**Table 5:** Interview report on nurse follow-up records for enteral nutrition (n = 22).

Data	Recorded		Not recorded	
	n	%	n	%
I record the amount of food and water given to patients.	216	100.0	0	0.0
I record the amount of gastric residual volume.	216	100.0	0	0.0
I record the date of nutrition tube insertion.	188	87	28	13
I record the change date of nutrition set.	157	72.7	59	27.3
I record the length of the protruding part of the Nutrition tube.	68	31.8	148	68.2
Nutrition tube number	0	0.0	100	100.0

In this study, all nurses were required to record the amount of food and water provided to the patients and the amount of residual gastric volume in the follow-up form. It was stated that 87% of the nurses should record the date of feeding tube insertion, 72.7% should record the feeding set-change date, and 31.8% should record the outside part of the feeding tube. None of the nurses stated that the number of feeding tubes should have been recorded.

## 4. Discussion

Although 90.9% of the nurses stated, “I change the feeding set daily” (Table 4) during the interview, 86.4% of the nurses changed the feeding set daily and wrote a date on it in bedside observations (Table 1). This situation is thought to be caused by nurses’ workload. Based on previous studies, the feeding set should be changed every 24 hour for infection.^[21]^

During the observation period, 77.3% of the nurses fixed their feeding tubes secure (Table 1). It was observed that 22.7% of the nurses fixed the irritated skin under the eyes and closed the nostrils. In all observations, nurses checked that the tube was in place before feeding. In the interviews, 27.3% of the nurses stated that “I give air with a syringe for tube control and listen to the stomach using a stethoscope." Sixteen nurses (72.7%) used the expression “I look at the gastric residue for tube control” (Table 4). Considering the evidence to guide nursing practice in confirming the location of enteral catheters, methods such as radiography, respiratory distress symptoms, visual content of aspirated fluid, pH measurement, auscultation, enzyme tests, the air bubble method, capnography, and gastric content extraction from the feeding tube have been used.^[11,13,24–28]^ For the evaluation, pH was considered a reliable method, and 0.2 to 1 mL of gastric juice is aspirated after the procedure and evaluated using pH test strips or paper. For correct placement, the pH should be 5.5 and below, but care should be taken if the pH is 6 or above.^[28,29]^ Turgay (2004) determined that gastric pH measurement was more effective than listening to a stethoscope in determining the tube location.^[18]^

*Gastric residue*: Before each feeding, the infant’s stomach was aspirated with an orogastric or Ng tube by a nurse to determine whether the food given in the previous feeding was tolerated or digested. The volume of nutrients left in the stomach after each feeding is a reflection of the time of gastric emptying, intestinal function, position of the baby, or feeding tube.^[29,30]^

For a baby who receives more than 50% of the volume that should be fully enterally fed while being fed in an intermittent bolus, if the residue is more than 50% of the last feeding, or if the residues checked in the last 3 feedings are between 30-50%, it is considered significant.^[29,30]^

In all bedside observations, it was observed that nurses performed gastric residual volume (GRV) control before feeding and recorded it on the nurse follow-up form. It was observed that 72.8% of nurses wrote daily data on the GRV injector (Table 1). GRV measurements are commonly used to determine food intolerance in enterally fed is GRV measurement.

In 93.1% of the observations, nurses raised the newborn’s head before enteral feeding, and in 88.8% of the observations, food was provided at the appropriate rate (Table 1). To prevent aspiration, it is necessary to raise the bed head of the patient to 30° to 45°, measure the gastric residual volume, not provide a large amount of food, and follow the patient closely.^[31]^

In the interview, 81.8% of the nurses said, “While giving the food, I pull the plunger of the syringe and wait for it to flow from above with negative pressure.” 18.2% of the nurses used the expression “I will put the syringe a the end of the tube and give the formula" (Table 4). For food drawn into the syringe to flow into the stomach with gravity drainage and negative pressure, it should be maintained 20 cm above the baby. Feeding food with a positive-pressure injector may cause perforation in newborn.^[4]^

Abdominal distension in enterally fed patients may develop because of factors such as the osmolarity of the given nutritional solution, the amount of fat it contains, the drugs used by the patient (opioids, etc), and gastric atonia. In addition, hot and cold feeding solutions also affected the development of distension. It is important to provide nutrients at appropriate rates to prevent distension. In addition, nurses measured the abdominal circumference of enterally fed patients using a tape measure, starting from the navel level, to determine the decrease or increase in distension.^[19]^

At the end of the interview with the nurses, all the nurses stated that “I wash the residue injector after looking at the gastric residue and leave it clean.” However, in 31.8% of observations, the injector was wrapped in a napkin and placed on the edge of the incubator without washing. The injector was washed and left clean in 68.2% of observations (Table 1). According to work and maintenance guidelines, the injector used to prevent the development of infection should be changed every 24 hour, and left clean by washing with water after each use.^[20]^

In all observations, after providing food and medicine, water was provided and the tube was cleaned. This application prevents clogging of the feeding tube.

The nurses recorded the amount of food and water provided and the GRV in the follow-up form.

The date of tube insertion was recorded in 87% of observations. The feeding set change date was recorded in 72.7% of observations. The length of the outer part of the tube was recorded in 31.8% of observations. However, the number of feeding tubes was not recorded during any of the observations or interviews (Tables 2 and 5). Studies have shown that different results are obtained in gastric residual volume measurements with feeding tubes of different numbers; reflux development is more common in tubes with large diameters, and obstruction develops more frequently in feeding tubes with small diameters.^[32–34]^ Nurses should record the number of feeding tubes required to evaluate the risk of developing such complications. In this study, the observation and interview reports of nurses’ follow-up records on enteral nutrition were consistent. During the interview, it was observed that the nurses recorded the points they knew about enteral nutrition that should be recorded during the observation, but did not record the points they did not know. Nurses lacked knowledge about issues that should be recorded regarding enteral nutrition. Incomplete records of nurse follow-up forms may also lead to complications. Complications such as esophageal rupture, pneumonia, apnea, gastric and duodenal rupture, pharyngeal perforation, and epistaxis may occur during enteral feeding.^[13]^ All the nurses in the study stated that aspiration and vomiting may be among the complications that occur during application. 90.9 of the nurses, 90.9% stated that NEC might be a complication that may occur during application. 54.5 of the nurses, 54.5% stated that perforation may be one of the complications that may occur during application. The risk of aspiration caused by incorrect insertion is one of the most common complications. The major complication risk of enteral nutritional status aspiration is 1% to 4%. Studies have shown that Many cases of pneumonia develop as a result of aspiration.^[35]^

As many complications associated with enteral feeding can develop when appropriate nursing care is not provided, the responsibility of nurses is primarily to prevent the development of complications, recognize and interpret the changes in the patient when they develop, and inform the physician by taking the necessary precautions. Nursing care, which plays a key role in the success of enteral nutrition, should facilitate nutrition, increase patient comfort, and reduce complications.^[16]^

### 4.1. Limitations of the research

The limitations of the study were the inability to make observations and interviews with nurses who had to work at night, as observation and interviews were allowed during daytime working hours in the neonatal intensive care unit. However, our study was planned by considering the number of nurses in the groups included in these limited studies, and the results obtained from the data obtained in our study had the same reliability as those of other studies.

## 5. Conclusion and suggestions

Although all nurses received enteral nutrition education in the undergraduate curriculum, during the observation and interview, there was a lack of knowledge during the interviews and observations. Due to the lack of knowledge on some issues, it was also observed that they could not correctly reflect the practices related to enteral nutrition correctly during the observations. It is recommended that nurses working in neonatal intensive care units regularly plan training, in which the results of evidence-based studies on enteral nutrition are shared. For the results of evidence-based studies on enteral nutrition to be used in practice, it is recommended to share the results of the studies in the literature with clinical nurses, and to conduct a study on this subject with different sample groups in different hospitals. It is recommended that an enteral nutrition care guide be prepared based on evidence from clinical studies.

## Author contributions

**Conceptualization:** Senay Cetinkaya.

**Data curation:** N. Ecem Oksal Gunes.

**Formal analysis:** N. Ecem Oksal Gunes.

**Funding acquisition:** Senay Cetinkaya, N. Ecem Oksal Gunes.

**Investigation:** Senay Cetinkaya, N. Ecem Oksal Gunes.

**Methodology:** Senay Cetinkaya.

**Project administration:** N. Ecem Oksal Gunes.

**Resources:** Senay Cetinkaya.

**Software:** N. Ecem Oksal Gunes.

**Supervision:** Senay Cetinkaya.

**Validation:** Senay Cetinkaya.

**Visualization:** Senay Cetinkaya.

**Writing – original draft:** Senay Cetinkaya.

**Writing – review & editing:** Senay Cetinkaya.
